# Designing an implementation intervention with the Behaviour Change Wheel for health provider smoking cessation care for Australian Indigenous pregnant women

**DOI:** 10.1186/s13012-017-0645-1

**Published:** 2017-09-15

**Authors:** Gillian S. Gould, Yael Bar-Zeev, Michelle Bovill, Lou Atkins, Maree Gruppetta, Marilyn J Clarke, Billie Bonevski

**Affiliations:** 1 School of Medicine and Public Health, University of Newcastle, University Drive, Callaghan, NSW 2308 Australia; 2 University College London, 1-19 Torrington Place, London, WC1E 7HB UK; 3 Clarence Specialist Clinic, 86 Through Street, South Grafton, NSW 2460 Australia

**Keywords:** Behaviour Change Wheel, Smoking cessation, Pregnancy, Health provider training, Indigenous populations

## Abstract

**Background:**

Indigenous smoking rates are up to 80% among pregnant women: prevalence among pregnant Australian Indigenous women was 45% in 2014, contributing significantly to the health gap for Indigenous Australians. We aimed to develop an implementation intervention to improve smoking cessation care (SCC) for pregnant Indigenous smokers, an outcome to be achieved by training health providers at Aboriginal Medical Services (AMS) in a culturally competent approach, developed collaboratively with AMS.

**Method:**

The Behaviour Change Wheel (BCW), incorporating the COM-B model (capability, opportunity and motivation for behavioural interventions), provided a framework for the development of the Indigenous Counselling and Nicotine (ICAN) QUIT in Pregnancy implementation intervention at provider and patient levels. We identified evidence-practice gaps through (i) systematic literature reviews, (ii) a national survey of clinicians and (iii) a qualitative study of smoking and quitting with Aboriginal mothers. We followed the three stages recommended in Michie et al.’s “Behaviour Change Wheel” guide.

**Results:**

Targets identified for health provider behaviour change included the following: capability (psychological capability, knowledge and skills) by training clinicians in pharmacotherapy to assist women to quit; motivation (optimism) by presenting evidence of effectiveness, and positive testimonials from patients and clinicians; and opportunity (environmental context and resources) by promoting a whole-of-service approach and structuring consultations using a flipchart and prompts. Education and training were selected as the main intervention functions. For health providers, the delivery mode was webinar, to accommodate time and location constraints, bringing the training to the services; for patients, face-to-face consultations were supported by a booklet embedded with videos to improve patients’ capability, opportunity and motivation.

**Conclusions:**

The ICAN QUIT in Pregnancy was an intervention to train health providers at Aboriginal Medical Services in how to implement culturally competent evidence-based practice including counselling and nicotine replacement therapy for pregnant patients who smoke. The BCW aided in scientifically and systematically informing this targeted implementation intervention based on the identified gaps in SCC by health providers. Multiple factors impact at systemic, provider, community and individual levels. This process was therefore important for defining the design and intervention components, prior to a conducting a pilot feasibility trial, then leading on to a full clinical trial.

## Background

Implementation science is the study of the methods to promote the systematic uptake of evidence-based practice into routine care to improve the quality and effectiveness of health services [1]. In this case, the challenge was to implement what is known to be effective for smoking cessation care (SCC) during pregnancy, into the context of health providers working with Indigenous women served by Aboriginal Community Controlled Health Services (ACCHS).

Indigenous smoking rates are high during pregnancy: in some communities, up to 80% of pregnant women smoke or use tobacco in another form [2]. In Australia, pregnant Indigenous women smoke at almost four times the rate of their non-Indigenous counterparts (45% compared to 12% in 2014) [3]. Smoking prevalence of pregnant Australian Indigenous women has been slow to decline, and cessation rates in pregnancy are half those of non-Indigenous counterparts [3].

Impediments to smoking cessation are complex in this setting and reveal individual-, community- and system-level factors [4]. A very high baseline prevalence of smoking among Indigenous families and communities is one factor that may jeopardise the capacity of individual people to quit [5]. Other important factors perpetuating Indigenous tobacco smoking in Australia include the detrimental impact of European colonisation causing dispossession, degradation and loss, and during which tobacco was introduced to many Indigenous communities for the first time; how Indigenous workers were often only paid in tobacco; government policies such as children being forcibly removed from parents (termed ‘the stolen generation’); and racism [2]. Tobacco smoking has become a norm and a social lubricant in many and diverse Australian Indigenous communities [2]. Despite these factors, more Indigenous smokers want to quit smoking than their general population counterparts but are less likely to succeed [6, 7]. Another important factor is the necessity to develop a strategy to address the needs of health professionals, who have been reported to seldom perform all the recommended steps for smoking cessation care for pregnant women [8, 9].

Theory-based interventions are recommended when designing complex approaches to behaviour change and aid in the specification of potentially active ingredients [10]. In defining the components of such an intervention, an analysis of the target behaviour is a key [11]. Smoking cessation in pregnancy, in particular for Indigenous populations, is an example where multiple factors have been identified that could be critical when designing targeted approaches to smoking cessation [4, 12]. A lack of evidence for successful smoking cessation interventions for pregnant Indigenous populations highlights the importance of understanding context when designing intervention components, and using a systematic approach to avoid implementation challenges [5].

### Evidence-based smoking cessation care

There is robust evidence that a combination of two approaches, the use of counselling and appropriate forms of pharmacotherapy, produces better outcomes than each alone in the general population of smokers [13]. Nicotine replacement therapy (NRT) is the most appropriate type of smoking cessation pharmacotherapy in pregnancy [14]. Emerging evidence from Cochrane Reviews indicates that counselling and NRT are effective in pregnancy [5], although these approaches have not yet been shown to be efficacious in Indigenous pregnancies. However, only two randomised controlled trials have been conducted globally in Indigenous pregnant women [15–17].

### The Indigenous Counselling and Nicotine QUIT in Pregnancy intervention

The Indigenous Counselling and Nicotine (ICAN) QUIT in Pregnancy is an evidence-based smoking cessation implementation intervention, developed primarily as a training intervention for health providers through webinar. It aims to support health providers to provide culturally targeted smoking cessation care to pregnant Indigenous smokers, attending Aboriginal Medical Services. Resources were developed to aid the implementation of the provider-patient consultation process (to aid counselling and provision of NRT) and included three 1-h interactive webinar sessions with PowerPoint presentations and short embedded videos, a training manual, a flipchart and a desktop guide (as a mouse pad). As part of the implementation, pregnant patients were to be provided a combined educational and motivational booklet with augmented reality videos within print media, and an educational and motivational video that could be watched in the clinic waiting room. Patient instructions for NRT were included in the patient information booklet by video and text. In addition, the implementation intervention provided free samples of NRT, and free courses of oral forms of NRT for the women to be prescribed at the services, and audit and feedback for the services about prescribing rates of NRT.

These resources developed through the process outlined below, went on to be reviewed by an expert panel and tested in several focus groups with health providers and Aboriginal women in three Australian states [18]. The resources then underwent a series of refinements [18]. Throughout this iterative process, extensive consultation and negotiation processes occurred, working closely with Aboriginal Medical Services and a Stakeholder and Consumer Aboriginal Advisory Panel [19].

In order to design the ICAN QUIT in Pregnancy implementation intervention and to address previous implementation challenges (revealed by similar trials for Indigenous women during pregnancy [15–17]), the Behaviour Change Wheel (BCW) [20] and the Theoretical Domains Framework (TDF) were used [21]. These models were used to design a service-level approach, which would address the evidence gaps and provide a targeted approach for smoking cessation care at the health provider (HP) and patient levels.

### Underpinning models and frameworks

#### Behaviour Change Wheel

**Fig. 1:**
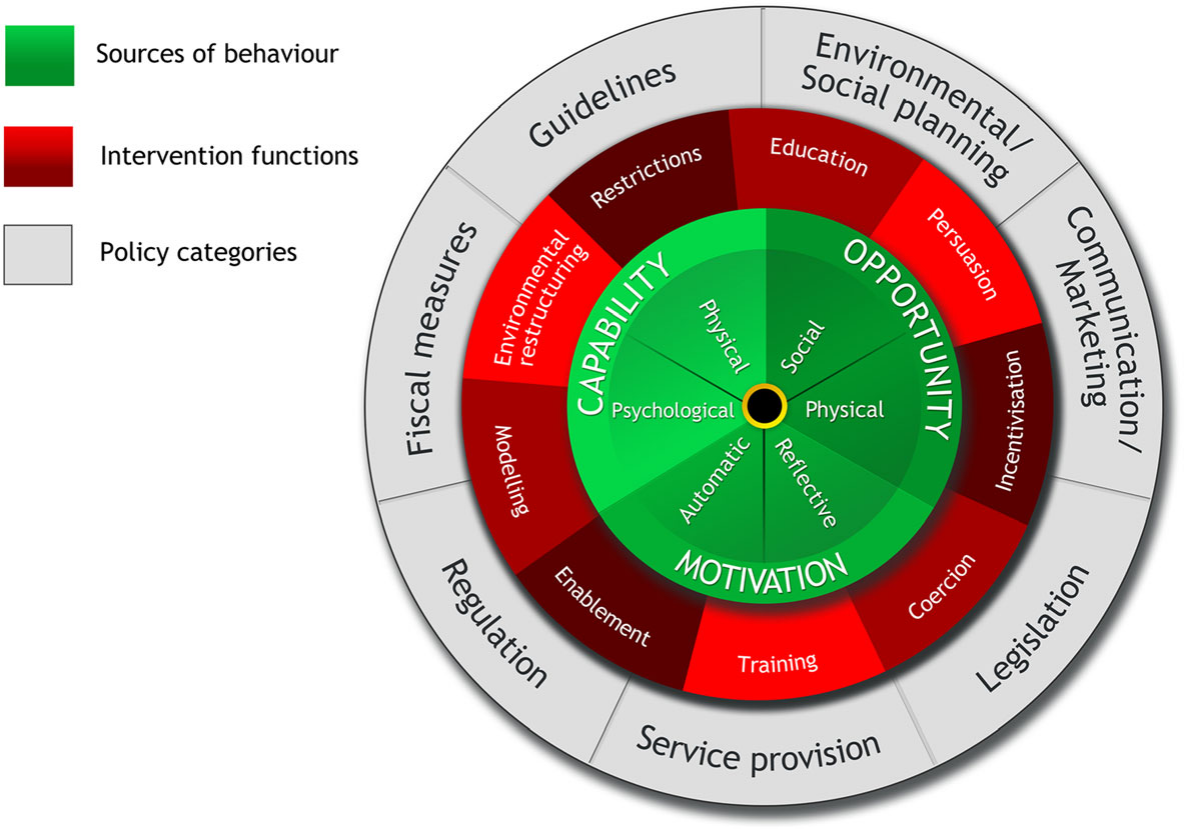
The Behaviour Change Wheel (reproduced with permission from authors) [11]

The BCW is a parsimonious model synthesising many behaviour change theories (see Fig. 1) [20]. At its hub is the COM-B model, which expands to capability (C), opportunity (O) and motivation (M)—these are all needed to produce or change a behaviour (B) [20]. The COM-B model recognises that behaviour is part of an interacting system involving an individual’s or group’s capability (physical and psychological), opportunity (social and physical) and motivation (reflective and automatic). A mid ring on the BCW comprises nine intervention functions, and on the outer ring, there are seven policy-level strategies. The BCW is used to link influences on behaviour, identified by the COM-B, to potential intervention functions and policy categories.

#### Theoretical Domains Framework

The TDF is an implementation science model comprising 14 domains that can categorise influences on behaviour [21]. These include the following: ‘Knowledge’, ‘Skills’, ‘Social/Professional Role and Identity’, ‘Beliefs about Capabilities’, ‘Optimism’, ‘Beliefs about Consequences’, ‘Reinforcement’, ‘Intentions’, ‘Goals’, ‘Memory, Attention and Decision Processes’, ‘Environmental Context and Resources’, ‘Social Influences’, ‘Emotions’ and ‘Behavioural Regulation’. According to Michie et al., links between the BCW and TDF can be utilised in the analysis and design of targeted interventions [11].

The BCW and TDF have been used previously to analyse health provider and patient or consumer behaviour; for example, Alexander et al. used the TDF and the COM-B to analyse barriers to the use of the Healthy Kids Check, introduced by the Australian Government in 2008 to guide the health screening of pre-school children by GPs and nurses [22]. Gould used the BCW to analyse barriers, enablers and strategies to improve smoking cessation care for pregnant Indigenous women [4].

The BCW is emerging as an important tool to rigorously develop interventions prior to clinical trials. The TDF and BCW have been used to systematically develop complex interventions in other populations and for other behaviours: several targeted health provider behaviour [23–28]. Sinnott et al. used the BCW to develop an intervention to improve medication management in multimorbidity by GPs, by uniquely developing a collaborative shared decision-making approach between peers [26]. McSharry et al. developed a multilevel intervention to increase delivery of sexual health counselling by cardiac rehabilitation staff [25]. Murphy et al. used the TDF and BCW to develop a capacity-building program to enhance pharmacists’ roles in mental health care [27]. The latter focused on health provider training, using a train-the-trainer approach. Similar to our approach, Murphy et al. highlight the iterative and fluid nature of the developmental process, which is challenging to capture. Relevant to our study, researchers applied the BCW and COM-B model at both the provider and patient levels to develop interventions to encourage long-term hearing aid use [24] and to encourage nurse-led physical activity in patients [21, 28]. More specifically for smoking cessation, for example, the BCW was used to develop a mobile phone App to encourage patients to use UK Stop Smoking Services, prior to a full trial of the intervention [29], and an App to support smoking cessation among pregnant women [30].

#### Indigenous-specific factors

In addition to the BCW and TDF, two factors appear to be relevant for developing Indigenous-specific approaches related to tobacco control and smoking cessation. A factor analysis of 47 organisations developing tobacco control messages for Indigenous Australians revealed two important, yet separate factors of ‘cultural understanding’ and ‘rigour’. Aboriginal Medical Services demonstrated strength in their cultural understanding while universities and government organisations demonstrated strength in rigour. A combination of cultural understanding and rigour was applied by few participating organisations. However, these two important factors emerging from the analysis can be seen as an opportunity to bring the best of two worlds together—Indigenous and Western viewpoints [31].

## Methods

### Aim, design and setting

The aim of this study was to describe (1) how implementation and intervention components for the ICAN QUIT in Pregnancy were developed for health provider and patient behaviour change based on the TDF and BCW and (2) comment on the translation of current evidence from smoking cessation care during pregnancy in conjunction with Indigenous researchers and an Aboriginal community advisory panel, and relevant to an Indigenous context.

This study was based on a growing body of evidence including systematic reviews by Okoli et al. about the provision of smoking cessation care by health providers [9] and by Baxter et al. about the uptake of smoking cessation care by pregnant women [26, 32], which demonstrated gaps in delivery of elements of SCC from the provider and patient viewpoints, and a systematic review by Gould et al. about Aboriginal women’s views of smoking during pregnancy [33]. Several narrative explorations of the influences on smoking among Indigenous women including two analyses using the BCW [2, 4, 34, 35], and empirical studies from both health providers’ and Indigenous women’s views about knowledge, attitudes and practices, were considered [36–40].

### Materials and processes

We conducted further research to refine our understanding about practices from the health provider view and the patients’ viewpoints. Thus, we based our initial intervention design on several studies. These included the following:

1. A systematic review of knowledge, attitudes and practices of health providers globally in providing smoking cessation care for pregnant women. The review, (currently being conducted) includes self-report from the health providers, observational studies and women’s reports of the care they received. Seventy-nine papers were included: 53 quantitative, 24 qualitative and 2 mixed methods. A meta-analysis was performed of pooled estimates of how often health providers perform each of the 5As (‘Ask’, ‘Advise’, ‘Assess’, ‘Assist’, ‘Arrange’), and prescribing rates of NRT. Qualitative data was extracted for an analysis of issues regarding smoking cessation care and the use of NRT, according to the BCW and COM-B, from the 24 qualitative and two mixed methods paper.
2. A systematic review of 23 included papers about interventions to improve health providers’ smoking cessation care for pregnant women, globally (in process)
3. A survey of 378 general practitioners (GPs) and obstetricians’ knowledge, attitudes and practices related to smoking cessation care for pregnant women. This included an analysis using the TDF and revealed several components of SCC that were less than ideal or missing, for example assisting pregnant women to quit and prescribing suitable forms of pharmacotherapy [8]. (Results are summarised below in the section on step 2 of the process.)
4. A qualitative study of 20 Aboriginal women who were pregnant or had recently given birth and were smoking or ex-smokers. The study focused on the women’s narratives of smoking before, during and after pregnancy and their attitudes and experiences of accepting smoking cessation care and pharmacotherapy [29, 41, 42].

Thus, we took into consideration multiple studies on Indigenous pregnancy from researchers in this field [12, 33, 36, 38, 39, 43–46], identifying the following major factors:

- Smoking by Indigenous women during pregnancy is a complex challenge and has multiple contributing factors.
- Clinicians ask and advise about smoking but less frequently assess, assist and arrange follow-up.
- Clinicians report a lack of confidence and optimism for helping their pregnant patients stop smoking. NRT prescription rates are also low.
- Aboriginal pregnant women report deficiencies in being provided important elements of smoking cessation care, such as being prescribed NRT, and being given consistent messages.

In the developmental phase of the implementation intervention, we considered approaches to modify both health provider behaviour, so that they more consistently provide evidence-based smoking cessation care to Indigenous women, and Indigenous women’s behaviour regarding their tobacco smoking practices during pregnancy. The health providers of interest were those working within the ACCHS in Australia. These included GPs, midwives, nurses, Aboriginal Health Workers and other relevant allied health professionals.

The research team was multidisciplinary and included Aboriginal and non-Aboriginal researchers from medical, social science, art, public health, behavioural science and education backgrounds. The team developed the resources collaboratively over several months and in consultation with a Stakeholder and Consumer Aboriginal Advisory Panel through a community-based participatory action research process [19]. The process of the intervention development, while guided by the stated theoretical frameworks, was iterative and fluid and evolved over several months of working within the core research team and the broader group of stakeholders.

### Use of the BCW and TDF by the research team

Using the BCW to translate the evidence into a culturally competent approach and guide the implementation intervention design, we followed the three stages recommended in Michie et al.’s “Behaviour Change Wheel” guide and associated worksheets [11]. Two of the team (GG and YBZ) attended a BCW training course run by the book’s author and our co-author (LA).

**Fig. 2:**
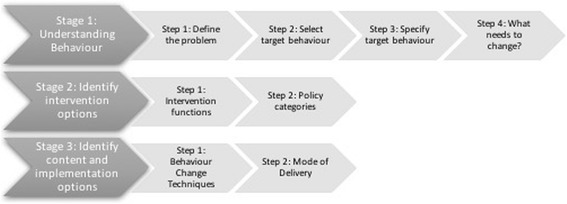
Stages and steps required to develop and implement an intervention according to the Behaviour Change Wheel. Adapted from Figure on page 31, Behaviour Change Wheel—a guide to designing interventions (with permission from the authors) [11]

Figure 2 shows the schema of the three stages and the composite steps in each, according to the BCW guide [11].

## Results

The results from this analysis are structured according to the recommended stages and steps for intervention design, as described by Michie et al., and designated in Fig. 2 [11].

## Designing approach to health provider behaviour change

### Stage 1: understand the behaviour

#### Step 1: define problem in behavioural terms

**Fig. 3:**
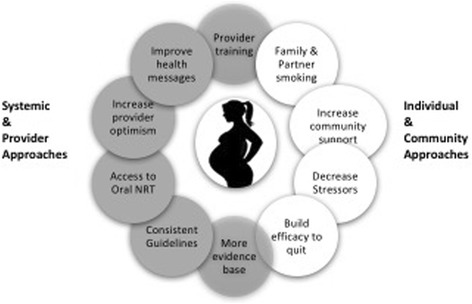
Approaches to improve smoking cessation among pregnant Indigenous women

A conceptual model (Fig. 3) depicts the multiple factors that influence smoking behaviours and smoking cessation among Indigenous women [2, 4, 12, 33–36, 38–40, 42, 44, 46–49]. These factors have an impact at the systemic level, provider level and community and individual levels. Although work is going on to attempt to remediate some of these factors (such as the policy-level lobbying to improve access and affordability of oral forms of NRT), this intervention is focused on translating the evidence-based practice for SCC in pregnancy to the Indigenous context.

#### Health provider behaviours

We identified the behavioural problem as a lack of essential elements in the health providers’ provision of evidence-based SCC for pregnant Indigenous women who smoke, such as prescribing NRT, providing cessation support, involving family members and following up. Health providers are required to provide SCC to pregnant women who smoke. The Australian RACGP guidelines recommend initially using supportive counselling, but if the woman cannot quit, she can be also offered NRT to assist cessation [50].

A systematic review by Okoli et al. [9] and our own systematic review (Gould 2017, unpublished data) revealed that while health providers often ‘Ask’ and ‘Advise’ about smoking cessation to pregnant women, they less commonly ‘Assist’ women to quit smoking. A lack of support from health providers for smoking cessation during pregnancy was cited as a barrier in previous research, with Aboriginal women and other vulnerable groups [41, 51].

The variety of health providers needing to improve access to SCC within the ACCHS is manifold. Depending on the stage of pregnancy, and the services themselves, women will see several different health professionals. We considered all health providers within a service would need to have the same over-arching approach for SCC, so the women would not receive conflicting advice. Thus, a whole-of-service approach was considered.

#### Step 2: select the target behaviour

Several evidence-based practices are recommended for SCC, including the 5A’s (‘Ask’, ‘Advise’, ‘Assess’, ‘Assist’ and ‘Arrange’) [50], the ABC (‘Ask’, ‘Brief advice’, ‘Cessation’) [11] [52] and the AAR (‘Ask’, ‘Advise’, ‘Refer’) [53]. Gould et al. proposed that health providers should take a holistic approach to SCC for pregnant Aboriginal women and recommended using an ABCD approach—A (Ask/assess), B (Brief advice), C (Cessation), D (Discuss psychosocial context) [43].

A survey of 378 GPs and obstetricians’ knowledge, attitudes and practices related to SCC for pregnant women revealed that clinicians seldom perform the 5As of smoking cessation (19.6% always/often) [8]. While more perform the AAR (49.2% always/often), there are few referral options for specialised services for pregnant women, and many are not being referred or followed up. Clinicians fairly reliably (over 75%) asked and advised about smoking cessation, but only a third actually assisted pregnant women to achieve smoking cessation. Cessation support was provided always by 33.6%; NRT was recommended/prescribed always by 11.1% and never by 25.1%. The TDF analysis revealed that lack of optimism, lack of time and lack of resources were the most frequently cited barriers [8].

Similarly, a survey specifically with health providers working with Aboriginal pregnant mothers revealed that only 4.7% recommended NRT to most or all pregnant clients who smoked [40].

Furthermore, Aboriginal women in our qualitative study revealed that they were seldom offered NRT, and when an offer did occur, it was as a ‘one-off’ offering, and the approach and messages were inconsistent [41]. Women reported they were often told to ‘cut down’ their smoking, but rarely supported to quit [41].

A review of this combined evidence led to a decision to primarily target the behaviour of health providers in offering NRT to assist smoking cessation. In other settings, assisting smokers to quit by providing a prescription of NRT was more effective than providing advice (RR 1.68, 95% CI 1.48–1.89 vs. RR 1.24, 95% CI 1.16–1.33) [41, 51]. A parallel target was to support the provision of a holistic culturally competent approach, taking into account the social determinants of health and psychosocial factors related to continued smoking during pregnancy, that is exemplified in the ABCD approach [43], and thus provide resources to target the patients’ needs also.

#### Step 3: specify the target behaviour

In specifying the target behaviour, the BCW guidebook recommends consideration of Who, What, When, Where, How often and with Whom. The target behaviour therefore would be for clinicians (Who) in the ACCHS (Where) to proactively offer assistance to every pregnant Indigenous smoker (Whom) to quit smoking (What), initially by counselling, but importantly by offering NRT, if the woman was not able to achieve abstinence in the first 2–3 days of a quit attempt (When). This would ideally be on every occasion that a smoker is seen (How often) until abstinence achieved. Although some guidelines suggest women should try a quit attempt unaided by pharmacotherapy for 2 weeks prior to be offering NRT, we considered this inappropriate in this context. It is notable that in Eades et al.’s RCT, women were asked to do this, and between the second and third visits, there was a dramatic drop-off in attendance from 70 to 30% of the cohort [15]. So as not to lose momentum in the quit attempt, and in the context of wanting women to quit as soon as possible in pregnancy, an expedited use of NRT was favoured [54].

#### Step 4: identifying what needs to change

Table 1 summarises the key items that required remediating for health providers, by considering the BCW, the COM-B model and the TDF. Key areas for performance improvement included capability (psychological skills), motivation (optimism) and opportunity (resources/time).

**Table 1:** Intervention components targeting health provider behaviour

Barriers to smoking cessation care	COM-B	TDF	Intervention function	BCTs	Translation of BCTs within the ICAN QUIT in Pregnancy intervention
Clinicians infrequently provide cessation support during pregnancy. Clinicians lack knowledge, skills and confidence to counsel women who smoke during pregnancy and to prescribe NRT. Lack of clinician training relevant to smoking cessation during pregnancy Women report infrequently receiving assistance from clinicians	Psychological capability	Cognitive and interpersonal skills	Education Training Enablement	Information on health, social, emotional and environmental consequences Information antecedents Instruction on how to perform behaviour	Webinar training on how to consult Indigenous pregnant smokers and prescribe NRT Training manual Videos of providers and patients
		Memory, attention and decision	Environmental restructuring	Restructuring physical environment Prompts, cues	Flipchart and desktop guide Patient resources
		Behaviour regulation	Modelling Incentivisation	Demonstration of behaviour Feedback on behaviour Rewarding completion	Audit and feedback about NRT prescribed CPD points for training
Clinicians lack optimism that their treatment will be successful during pregnancy	Reflective motivation	Belief about capability Belief about consequences Optimism	Education Training Persuasion Enablement	Information on health, social, emotional and environmental consequences Credible source Persuasion about capability Framing/reframing Salience of consequences Social comparison Adding objects to the environment	Provide resources Smoking reframed as an addiction, not a choice Inform re standard practices and evidence-based practices Building self-efficacy Build response efficacy—it is worthwhile—NNT only 16–17 for quitting Motivational videos, testimonials and success stories Celebrating small wins and turning ‘near misses’ into success
	Automatic motivation	Reinforcement Emotion	Environmental restructuring Persuasion	Credible source Exposure Framing/reframing Social comparison	Provide resources Emotive videos of health providers and patients
Clinicians lack time and resources to provide smoking cessation care. Oral NRT is not subsidised in Australia forming a barrier to prescribing	Physical opportunity	Environmental context Resources	Environmental restructuring Enablement	Adding objects to the environment Problem solving Self-monitoring of behaviour	Free NRT samples and oral NRT vouchers Referral pads Flipchart and desktop guide Patient booklets
Few clinicians perform comprehensive smoking cessation care so there are few role models	Social opportunity	Norms Social influences	Modelling Education and training	Social comparison Credible source Instruction on how to perform behaviour Self-monitoring of behaviour	Whole of service training Interactive webinar Audit and feedback Videos of positive attitudes of other health providers

*Please note:* physical capability not targeted; *BCT* behaviour change technique; *COM-B* capability, opportunity, motivation-behaviour; *ICAN QUIT in Pregnancy* Indigenous Counselling and Nicotine QUIT in Pregnancy

According to the BCW, for this to be successful, clinicians would be required to increase their psychological capability, i.e. knowledge and skills, and reflective motivation, i.e. confidence and optimism, in how to prescribe NRT for a pregnant Indigenous woman who smokes. Lower levels of confidence for NRT prescribing, compared to counselling, were revealed in our national study [8]. Clinicians also reported low optimism that their treatment would be effective [53]. Thus, providers need to build both self-efficacy and response efficacy. Furthermore, as the preferred oral forms of NRT to use in pregnancy are not subsidised in Australia, health providers require improvements in their physical opportunity to provide care, i.e. access to oral NRT supplies, in order to effectively prescribe [47]. Clinicians understanding that these practices are routine in other countries (such as in Australian guidelines and programs and in other countries) may help them improve their social opportunity.

### Stage 2: identify intervention options

#### Step 1: intervention functions

Table 1 also outlines the intervention components developed for health providers according to the analysis. Intervention functions which best met the APEASE (Affordability, Practicability, Effectiveness and Cost-effectiveness, Acceptability, Side effects/safety, Equity) criteria were considered for inclusion [11]. APEASE is a criterion for making context-based decisions on intervention content. Clinicians in our survey indicated several options to improve their smoking cessation care (Gould 2017, unpublished). Training was an option that most of these clinicians agreed on. This is supported by a Cochrane Review that found training of HPs in smoking cessation care increased abstinence in their patients [55]. Therefore, education and training were chosen as the predominant intervention function. However, to address the low optimism, we include persuasion, modelling and enablement in the intervention. Resources were also developed for the women to support health providers in their consultations, and to provide resources for health education, and practical assistance for their patients (described below).

#### Step 2: policy categories

On the policy layer of the wheel, service provision and guidelines were our main policy targets. We wanted to aim our approach to the whole of the ACCHS, in recognition that many health providers and allied health professions have a role in consulting with Indigenous women during their pregnancy. We wanted smoking cessation to be ‘everyone’s business’. Furthermore, we developed a comprehensive treatment manual that gave very practical guidance for the approach, based on the published pragmatic guide and the RACGP guidelines [43, 50, 56].

### Stage 3: identifying content and implementation options

#### Step 1: behaviour change techniques

Table 1 also specifies the behaviour change techniques (BCTs) related to the above analysis and to address each intervention function. A taxonomy of BCTs was developed by Michie et al. (BCTTv1) as a comprehensive resource for intervention development [57]. Each of the 93 consensually agreed, distinct BCTs in the taxonomy are catalogued and described in detail. The BCTs identified for the ICAN QUIT in Pregnancy implementation intervention were those which authors considered as promising to elicit behaviour change in the health providers.

In summary, to implement this smoking cessation intervention optimally, we aimed to improve capability by training clinicians in NRT prescribing, structuring the consultation using a flipchart and prompts and regulating behaviour through audit and feedback (allowing social opportunity). To improve optimism, in the training, we present recent evidence about NRT and positive testimonials from patients and clinicians. We recognised that individual clinicians may not have experienced intervention success but needed to be exposed to that success vicariously through other’s testimonials, so they see that success is possible and worthwhile persisting for (response efficacy).

#### Step 2: mode of delivery

To accommodate challenges around time for health providers, the large geographical area of Australia and the limited resources of the research, we decided to trial training via interactive webinar. Webinar was chosen as a mode of delivery with the potential to increase the reach of health provider training to urban, rural and remote locations in Australia and improve the potential scalability. Although webinar is now a very common method of distance training, there is little research in the peer-reviewed literature about the efficacy or effectiveness of this method. However, we considered webinars to be affordable, cost-effective, safe, likely to be acceptable and highly equitable. They give the potential for face-to-face contact.

Training of short duration (40 to 120 min) has been shown to increase smoking session outcomes in patient outcomes in a Cochrane Review [55]. Thus, three 1-h webinar sessions were planned to bring the opportunity of training to services and accommodate time and location constraints, which may otherwise limit attendance by the health providers. Webinar sessions were to be interactive and include PowerPoint presentations, short videos and group discussions. These went on to be trialled in the pilot study in six services in three states [58].

### Design approach to patient resources

A parallel analysis was conducted to that described above for the health providers, to address behaviour change for pregnant patients. It was considered important to provide accompanying patient resources to support Indigenous women in their smoking cessation journey that would also accommodate variable literacy levels in this population. A lack of resources was also identified by health providers. Similar to Table 1 for the health providers, Table 2 outlines the intervention components and behaviour change techniques we developed for the pregnant patients who smoke, according to the parallel analysis.

**Table 2:** Intervention components targeting patient behaviour

Barriers to smoking cessation care	COM-B	TDF	Intervention function	BCTs	Translation of BCTs within the ICAN QUIT in Pregnancy intervention
Indigenous pregnant women report symptoms of nicotine dependence and withdrawal effects from attempts to quit. Increased nicotine metabolism in pregnancy can increase cigarette consumption and also requires higher doses of NRT	Physical capability	Physical skills	Education Enablement	Provide feedback on current behaviour and dependence levels Assess withdrawal symptoms Biofeedback with carbon monoxide readings Making a quit plan and/or setting quit date Advise on stop-smoking medication Enable clients to obtain free medication	Free NRT for physical addiction Videos on how to use different types of NRT
Aboriginal women lack detailed knowledge about the harms of smoking. Stressful life circumstances may also impact on a women’s psychological capability to quit. Historical antecedents of smoking in Indigenous communities, racism, health disparities and low socio-economic status can impair capability to quit	Psychological capability	Knowledge	Education	Provide information on consequences of smoking and smoking cessation Instruction on how to quit smoking Offer appropriate written materials	Health booklet, supportive counselling and videos showing effects of smoking on mother and child Discuss psychosocial contexts of smoking
		Cognitive and interpersonal skills	Persuasion	Facilitate goal setting Facilitate barrier identification and problem solving Facilitate relapse prevention and coping Facilitate action planning and develop quit plan Advise on conserving mental resources	Discussion of psychosocial context of smoking Build self-efficacy for quitting Culturally appropriate colouring-in pages for diversion and relaxation
		Memory, attention and decision	Environmental restructuring Enablement	Advise on avoiding social cues for smoking Elicit client views Provide reassurance	Text and video on how to make a smoke-free home Personalised quit plan and goal setting Patient resources
		Behaviour regulation	Enablement Modelling		Messages from salient others—peers and experts NRT to reduce withdrawal effects Carbon monoxide readings Videos of role models Counselling on stressors and triggers
Few positive role models, as Indigenous smoking prevalence is high Targeted messages preferred Existing media messages may lack salience. Not wanting to be ‘told what to do’. Didactic counselling styles are unwelcome	Reflective motivation	Social role/identity Belief about capability Belief about consequences Intentions Goals Optimism	Persuasion Education Enablement Incentivisation Modelling	Credible sources for messages Explain the importance of abrupt cessation Boost motivation and self-efficacy Rewards contingent on effort or progress Emphasise choice	Targeted salient messages Build self-efficacy Building response efficacy—stopping smoking is worthwhile Smoking as an addiction Link nicotine withdrawal and symptoms of ‘stress’ Emphasising choice to quit Resources and support Success stories and role models via videos Goal setting, quit plan and quit date Dealing with challenges
Change of role on becoming pregnancy positively reinforces need to quit. Protective attitudes to baby Cravings can impair motivation	Automatic motivation	Reinforcement			Self-rewards in quit plan Celebrating small wins Free NRT ameliorates withdrawal symptoms
Lack of optimism for quitting		Emotion	Environmental restructuring Persuasion Enablement		Addressing challenges in quit plan Emotive videos Free NRT ameliorates withdrawal/stress ﻿symptoms
Lack of access to services or presenting late to antenatal care Lack of targeted resources Lack of subsidised NRT Health providers not frequently offering assistance to quit	Physical opportunity	Environmental context Resources	Environmental restructuring Enablement Education	Advise on environmental restructuring Advise on changing routine	Trained providers to support their quit attempts Referrals to other services Flipchart Patient booklets
Few role models who have quit during pregnancy	Social opportunity	Social influences	Modelling	Provide normative information about others’ behaviour and experiences Advise on/facilitate use of social support	Involving family members Making a smoke-free home Increasing social support Positive peer role models through video stories

*BCT* behaviour change technique; *COM-B* capability, opportunity, motivation-behaviour; *ICAN QUIT in Pregnancy* Indigenous Counselling and Nicotine QUIT in Pregnancy

In developing the appropriate messages within the women’s resources, we deliberately included elements of both surface and deep structure to ensure cultural sensitivity. Resnicow et al. proposed that for health messages to be effective and culturally sensitive [59], attention should be paid to surface structure, i.e. the look of the messages, colours, graphics and people to enable a good fit, and so people from a group will be engaged and know the message is for them. Additionally, deep structure is needed to incorporate the deeper values of a group, such as cultural and family values, and important shared concerns and meanings: this governs the salience of the messages [59]. In a survey of Australian organisations developing tobacco control messages, deep structure was an important factor in cultural understanding, the use of which was characterised by Aboriginal Medical Services [31].

Having salient people to deliver the messages was vital, so we invited an Aboriginal obstetrician (Dr. Marilyn Clarke) and a Torres Strait Islander GP (Dr. Karen Nicholls) to deliver the health messages. Dr. Clarke explains how smoking affects babies in utero, and Dr. Nicholls instructs women on how to correctly use various forms of NRT. In addition, we included videos from peers about triggers for smoking and how to make a smoke-free home.

## Discussion

This study described the developmental and translational research to identify key components for a culturally competent smoking cessation implementation intervention aimed at educating and training health providers and providing resources to support Indigenous women to quit smoking and support making a smoke-free home. The BCW has been used previously to analyse and recommend targeted strategies [4, 35]. We provided a more detailed analysis of the theoretical components for a smoking cessation implementation intervention for pregnant Indigenous Australian women, than hitherto published, aimed principally at changing health provider behaviour. Patten developed a smoking cessation intervention for pregnant Alaska Native women after analysing community needs and preferences and based on the social cognitive theoretical framework [17]. Gilligan conducted an analysis of Indigenous smoking during pregnancy with the PRECEED-PROCEED model [60], prior to Eades et al. consulting with general practitioners, health care workers and community representatives in the health services to develop an intensive intervention for pregnant Aboriginal and Torres Strait Islander smokers [15]. Neither study published a detailed analysis about the theoretical framework used to develop the intervention.

Several papers have outlined how to optimise smoking cessation interventions aimed at vulnerable target groups [2, 12, 43, 48, 49, 51, 61–64]. For other conditions, there has been discussion on theory informing the development of Indigenous health programs. In an analysis of the development of anti-tobacco messages for Indigenous Australians, 55% (*n* = 26) of organisations reported using some type of theory, as one component of a more rigorous approach [31]. However, we believe this is the first time the BCW and TDF have been applied to the context of an Indigenous smoking cessation implementation intervention.

The benefit of using the BCW is that it encourages intervention designers, like us, to comprehensively and broadly consider options to intervene and then systematically select those that are most promising for the context. It aids in making the best use of the understanding and resources available to arrive at a behaviour change intervention [65].

### Strengths and limitations

This study strengthens the theoretical foundations on which to develop smoking cessation implementation interventions for Indigenous peoples. It brings together two important factors of cultural understanding and rigour [31] and applies the TDF and the BCW to the context of Indigenous smoking during pregnancy. A limitation is the iterative nature of the process: it is hard to capture the stepwise approach described by Michie et al. as it can be bi-directional at times, and resulting consultations within and without the core team meant that earlier stages may be revisited, and these may not be clearly documented. However, the Indigenous consultation and community-based participatory research process have been described in detail elsewhere [19]. The implementation intervention of the ICAN QUIT in Pregnancy will be tested in the pilot study and be adjusted as required [58].

### Implications for future research

These chosen intervention functions, mode of delivery and resources are in the process of being trialled for feasibility in a pilot study in six ACCHS, prior to it being implemented in a full trial [58, 66]. After this pilot study, end-users will be surveyed and interviewed to determine whether the intervention and study design need any modifications before preparing for a larger cluster randomised trial (renamed SISTAQUIT™—Supporting Indigenous Smokers To Assist Quitting). The systematic approach for the intervention development ﻿we have described﻿ will help streamline this process.

## Conclusion

Smoking during pregnancy contributes significantly to the health gap for Indigenous Australians. Multiple contributing factors impact at systemic, provider, community and individual levels. The ICAN QUIT in Pregnancy pilot implementation intervention used webinar to train health providers in Aboriginal Medical Services in a culturally competent approach that includes counselling and the use of nicotine replacement therapy for their pregnant patients who smoke. It includes culturally targeted resources appealing to Indigenous women that can engage and also account for low literacy by including embedded videos in print media. Using the BCW and TDF aided in scientifically and systematically informing a targeted intervention based on the identified gaps in SCC by health providers. This process was important for defining the design and intervention components, prior to conducting a pilot feasibility trial and then leading on to a full clinical trial.

## Abbreviations

BCT: Behaviour change technique
BCW: Behaviour Change Wheel
COM-B: Capability, opportunity, motivation, behaviour
GP: General practitioner
ICAN QUIT in Pregnancy: Indigenous Counselling and Nicotine QUIT in Pregnancy
NRT: Nicotine replacement therapy
RACGP: Royal Australian College of General Practitioners
RCT: Randomised controlled trial
SCC: Smoking cessation care
TDF: Theoretical Domains Framework
